# Human and Porcine Transmission of *Clostridioides difficile* Ribotype 078, Europe

**DOI:** 10.3201/eid2709.203468

**Published:** 2021-09

**Authors:** Geraldine Moloney, David W. Eyre, Micheál Mac Aogáin, Máire C. McElroy, Alison Vaughan, Tim E.A. Peto, Derrick W. Crook, Thomas R. Rogers

**Affiliations:** Trinity College Dublin, Dublin, Ireland (G. Moloney, M. Mac Aogáin, T.R. Rogers);; University of Oxford, Oxford, UK (D.W. Eyre, A. Vaughan, T.E.A. Peto, D.W. Crook);; Central Veterinary Research Laboratory, Celbridge, Ireland (M.C. McElroy);; St. James’s Hospital, Dublin (T.R. Rogers)

**Keywords:** Clostridioides difficile, Clostridium difficile, bacteria, transmission, humans, pigs, farms, community-associated infection, hospital-associated infection, genomics, inpatients, One Health, phylogeny, ribotyping, ribotype 078, zoonoses, Europe

## Abstract

Genomic analysis of a diverse collection of *Clostridioides difficile* ribotype 078 isolates from Ireland and 9 countries in Europe provided evidence for complex regional and international patterns of dissemination that are not restricted to humans. These isolates are associated with *C. difficile* colonization and clinical illness in humans and pigs.

*Clostridioides (*formerly *Clostridium) difficile* was considered to be a predominantly nosocomial pathogen until findings of several whole-genome sequencing studies suggested a more complex epidemiology. For example, Eyre et al. reported that only 35% of nosocomial *C. difficile* infections (CDIs) were potentially attributable to other cases on the basis of genomic data, and only 19% were additionally linked through sharing possible hospital-based contact (*1*). This finding suggests that a major proportion of *C. difficile* from CDI cases occurring in healthcare institutions originates from other sources, including the community (*2*).

Community-associated CDI (CA-CDI) is now well recognized, accounting for ≈25% of cases in Australia, <25% of cases in Europe, and 33% of cases in the United States (*3*,*4*). There is increasing recognition that *C. difficile* is a near ubiquitous environmental organism and that humans have widespread environmental exposure to it. *C. difficile* has been detected in samples from parks (24.6%); water sources, including rivers, lakes, and sea water; homes (17.1%); commercial stores; and other premises (6.5%–8.1%), in addition to hospitals (16.5%) (*5*,*6*). Isolates of *C. difficile* from these studies underwent ribotype analysis. Overall, ribotype 027 isolates were most commonly identified in hospital samples, and ribotype 014–020 isolates predominated in other environmental samples. Isolates of the most common ribotypes were not restricted to any particular location (*5*). These findings support the possibility that there are different sources for exposure to each *C. difficile* ribotype.

Occurrence of CDI caused by *C. difficile* ribotype 027 has been greatly reduced in the United Kingdom, most likely the result of the combination of antimicrobial stewardship and hospital infection prevention and control measures. However, these interventions have not reduced the incidence of infections caused by other ribotypes, including ribotype 078 (*7*).

Findings of genomic analysis of isolates from the European, Multi-Center, Prospective, Biannual, Point-Prevalence Study of *Clostridium difficile* Infection in Hospitalized Patients with Diarrhea (EUCLID) showed that specific *C. difficile* ribotypes were associated with healthcare clusters, and other ribotypes had an international distribution across Europe (*8*). For example, ribotype 078 isolates did not cluster by their country of origin, indicating a complex distribution unrelated to nosocomial transmission. The mechanisms of transmission have not been identified, but might be related to the movement of food, other animal-derived products, or persons across Europe (*8*).

*C. difficile* carriage and infection has been well described in livestock and other animals (*3*); certain ribotypes of *C. difficile* are considered to be major ribotypes from a One Health perspective. These ribotypes include ribotype 078, carriage of which has been reported in 9%–100% of piglets from North America, Europe, Asia, and Australia (*3*). Carriage rates in calves (56%) and cows (13%) have been lower. Although many studies did not identify any major carriage in adult pigs, 1 study in the Netherlands reported a rate ranging from 6.6% to 100% (*3*).

We have reported *C. difficile* ribotype 078 in cases of typhlocolitis in neonatal piglets in Ireland (*9*), and Knetsch et al. found that ribotype 078 isolates carried by farmers in the Netherlands and their pigs were identical by whole-genome sequence analysis (*10*). These findings suggest that *C. difficile* isolates might be shared between humans and pigs when in close proximity. However, the mechanisms and directions of transmission are not known.

In this study, we investigated the genomic relationships between *C. difficile* ribotype 078 isolates of human and porcine origin collected from Ireland and compared these with international ribotype 078 isolates. We also investigated the extent to which geographic proximity could explain clusters of clonal isolates.

## Methods

### Samples and Settings

Clinical isolates of *C. difficile* ribotype 078 were collected prospectively as part of an investigation of consecutive episodes of CDI conducted at St. James’s Hospital (Dublin, Ireland), a 900-bed tertiary referral center, during 2013–2016. Stool samples, sent from patients with diarrhea, had the *C. difficile* toxin B gene identified by using the EntericBio PCR Kit (Serosep, https://www.serosep.com). We reviewed medical notes of inpatients to obtain relevant clinical data, including antimicrobial drugs and proton pump inhibitors prescribed before the onset of diarrhea, features indicative of severe CDI with or without complications, and the antimicrobial drugs used for management of CDI. These data were pseudonymized and stored in a dedicated database.

We retrieved an additional 9 *C. difficile* 078 isolates from a study of recurrent CDI at St. James’s Hospital during 2012–2013 (*11*). Five additional *C. difficile* ribotype 078 isolates were provided from those submitted to a national surveillance study of CA-CDI in Ireland conducted during 2015. Isolates of *C. difficile* were recovered from pigs that had been referred for autopsy at the Central Veterinary Research Laboratory (CVRL; Backweston, Ireland) during 2014–2015, irrespective of the suspected cause of death, by sampling colonic contents or feces that had positive results for *C. difficile* toxins A/B by using the Premier Elisa Kit (Meridian BioScience Inc., https://www.meridianbioscience.com). We treated human fecal and porcine colonic/fecal samples with ethanol shock before anaerobic incubation on cycloserine cefoxitin egg yolk medium. DNA was extracted from resulting colonies for PCR ribotype analysis and Illumina (https://www.illumina.com) genomic library preparation as described (*11*).

### Whole-Genome Sequencing

Whole-genome sequencing was performed either on an Illumina MiSeq or MiniSeq platform at Trinity College (Dublin, Ireland) or on the Illumina HiSeq platform at the Wellcome Centre for Human Genetics, University of Oxford (Oxford, UK). Sequence data generated have been deposited in the National Center for Biotechnology Information Short Read Archive (https://www.ncbi.nlm.nih.gov/sra) under BioProject PRJNA692997.

We mapped sequence reads to the ribotype 078 reference genome M120 (GenBank accession no. FN665653.1), and identified high-quality variants by using an approach developed and calibrated for *C. difficile* (*1*) with later refinements (*12*) (Appendix). We obtained published comparison sequences from the EUCLID pan-European cross-sectional survey conducted during in 2012–2013 (*8*) and from farm animal and human isolates from the Netherlands (2002–2011) described by Knetsch et al. (*10*).

### Sequence Comparisons

We compared sequences by using single-nucleotide polymorphisms (SNPs) and obtained differences between sequences from maximum-likelihood phylogenies corrected for recombination (Appendix). We reviewed phylogenetic analysis of closely related genomes in conjunction with available epidemiologic data. Within the clinical database, CDI recurrence was defined as identification of 2 isolates within 10 SNPs from 1 patient (*1*) for which that patient had clearly documented clinical resolution of symptoms after their first episode. On the basis of rates of *C. difficile* evolution and within-host diversity (*1*), we defined plausible, short-term, transmission/mutual exposure as isolates differing by 0–2 SNPs.

We made epidemiologic matches between patients who had in-patient admissions and demonstrable links with respect to time, location, or healthcare staff, where their *C. difficile* isolates were within 0–2 SNPs. Because epidemiologic details were not available for either the CA-CDI investigation in Ireland or the EUCLID isolates, we analyzed linkage between cases on the basis of genetic similarity alone. These genomic pairs were named by the isolate sources in chronologic order of identification.

### Ethics

Investigation of hospital-associated CDI (HA-CDI) cases at St James’s Hospital was conducted after obtaining approval from the St. James’s Hospital/Tallaght Research Ethics Committee. Porcine isolates were exempt from requiring ethics approval.

## Results

A total of 171 *C. difficile* ribotype 078 isolates were included in the analysis: 53 isolates from CDI episodes in 44 inpatients at St. James’s Hospital, including 5 community-associated isolates; 20 porcine isolates from Ireland; 67 clinical, farmer, and porcine isolates from the Netherlands; and 31 clinical EUCLID isolates. We provide details of their country of origin, source, and date of isolation (Table 1). The EUCLID isolates were obtained from 9 countries in Europe. Six countries, including Ireland, submitted >2 isolates.

**Table 1:** Countries from which *Clostridioides difficile* 078 isolates originated, their identified sources, and date of collection*

Origin and source of isolates	Timeframe of collection	No. isolates
Ireland (*11*)		
HA-CDI	2012‒2016	48†
Porcine	2014–2015	20
CA-CDI	2015 Apr–Jun	5
Netherlands (*10*)		
CDI	2002–2011	31
Porcine	2009, 2011	20
Healthy farmers	2011	16
EUCLID (*8*), HA-CDI	2012 Dec‒2013 Aug	
Germany		9
Italy		7
United Kingdom		4
France		3
Portugal		3
Ireland		2
Spain		1
Greece		1
Austria		1

*CDI, *C. difficile* infection; EUCLID, European, Multi-Center, Prospective, Biannual, Point-Prevalence Study of *Clostridium difficile* Infection in Hospitalized Patients with Diarrhea; HA-CDI, hospital-associated CDI. †Includes 9 isolates from HA-CDI cases (*11*).

Of the 53 isolates causing CDI in Ireland, 9 were from recurrent CDI episodes in 7 patients (7 subsequent isolates were 0 SNPs different from, the baseline isolate, 1 was 1 SNP different, and 1 was 8 SNPs different). Only the first isolate from each patient was considered in subsequent analyses. We provide genomic relationships between the remaining 162 ribotype 078 isolates (Figure). Despite the diverse sampling frame, only limited diversity was seen; the greatest root-to-tip distance in the phylogenetic tree was 48 SNPs.

**Figure:**
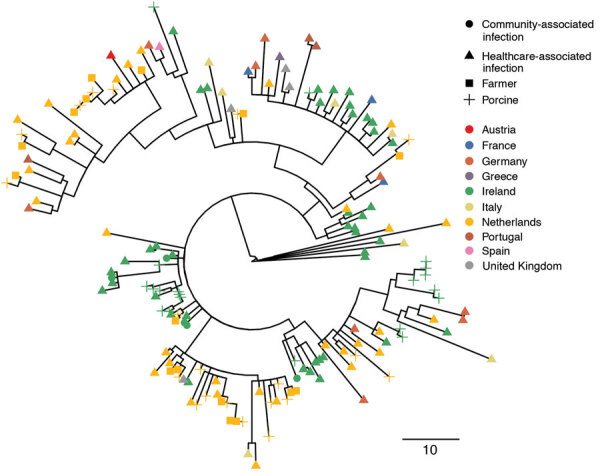
Recombination-adjusted maximum-likelihood phylogenetic tree of sequences from human and porcine *Clostridioides difficile* isolates from Ireland and 9 other countries in Europe. Isolates are shown as triangles for healthcare-associated *C. difficile* cases and circles for community-associated *C. difficile* cases. Isolates from pigs are shown as crosses and those from farmers as squares. The color at each tip indicates the country of origin of the isolate. The tree was based on 4,861 variable sites before correction for recombination, based on a median (interquartile ranges) of 93.4% (93.0%–93.8%) and (83.1%–96.2%) of the reference genome being called. Scale bar indicates single-nucleotide polymorphisms.

Isolates from Ireland were found throughout the tree, but specific clusters of these isolates were seen, including, as shown at the ≈240° (≈8 o’clock) position (Figure), a cluster of cases that included isolates from HA-CDI and CA-CDI cases as well as cases from pigs. Within this cluster, several porcine isolates were directly ancestral to 1 HA-CDI case. Another 5 CDI cases, including 1 CA-CDI, had another porcine isolate directly ancestral. This finding suggests a porcine origin for these cases, either directly or by >1 or more intermediate (unsampled) transmission routes. This same cluster also contained an isolate from a pig and a farmer from the Netherlands. Several other clinical isolates from the Netherlands were closely related to porcine isolates (Figure).

We provide epidemiologic links between genetically related isolates within 0–2 SNPs (Table 2). Although nearly all genomic pairs occurred among isolates with the same country of origin, the epidemiologic information available can explain only a small proportion of transmissions/mutual exposures.

**Table 2:** Pairs of *Clostridioides difficile* ribotype 078 isolates matched by country of origin and source case, with associated epidemiology*

Country	Source of isolate(s)	Country 2	Source of isolate(s)	No. pairs of isolates	Associated epidemiology
Ireland	CA-CDI	Ireland	CA-CDI	2	No known links
Ireland	CA-CDI	Ireland	HA-CDI	2	No known links
Ireland	HA-CDI	Ireland	HA-CDI	10	Possible transmission 6 pairs,† unknown for 4 pairs
Ireland	Porcine	Ireland	HA-CDI	3	No known links
Ireland	Porcine	Ireland	Porcine	12	8 pairs at 1 farm, 3 pairs at 1 farm, 1 pair at 1 farm, no pairs between farms
Ireland	CA-CDI	Italy	HA-CDI	1	Unknown
Ireland	HA-CDI	United Kingdom	HA-CDI	1	Unknown
Germany	HA-CDI	Germany	HA-CDI	1	Unknown
Netherlands	HA-CDI	Netherlands	HA-CDI	1	Unknown
Netherlands	CDI	Netherlands	Farmer	1	No known links
Netherlands	CDI	Netherlands	Porcine	1	No known links
Netherlands	Farmer	Netherlands	Farmer	3	Unknown
Netherlands	Farmer	Netherlands	Porcine	10	Farm exposures
Netherlands	Porcine	Netherlands	Porcine	1	No known links
Portugal	HA-CDI	Portugal	HA-CDI	1	Unknown

*CA-CDI, community-assocated *C. difficile* infection; HA-CDI, healthcare-associated *C. difficile* infection. †The 6 possible healthcare-associated transmission pairs shared time and space on the same hospital ward (n = 3) or time on different hospital wards while under the care of the same medical team (n = 3).

## Discussion

Our findings support a complex regional and international distribution of *C. difficile* ribotype 078 isolates. In contrast to the EUCLID study, which obtained samples on single days in winter and summer, more dense sampling was undertaken in our study. In the EUCLID study, no evidence of clustering of ribotype 078 within countries was seen, which is consistent with a complex pattern of dissemination in Europe over timescales spanning years (Figure). However, our study showed evidence of sublineages of ribotype 078 that are predominantly found in isolates from the Netherlands and others predominantly found in isolates from Ireland (Figure). It is likely that this denser sampling has enabled recent, local, onward transmission to be better captured. We also identify a EUCLID isolate from Italy (2013) and a CA-CDI isolate from Dublin, Ireland (2014), that are within 2 SNPs, which is consistent with a temporally related transmission. However, we do not know of any epidemiologic link between these 2 cases.

For 10 pairs of isolates within 2 SNPs from inpatients who had HA-CDI, possible healthcare-based epidemiologic links could be made for 6 of these pairs but not the other 4. Plausible ward-based transmission only accounted for 3 pairs. For other genetically related isolates pertaining to inpatients in our study, there was a median of 559 days between their associated CDI episodes (range 147–651 days) without overlapping hospital admissions or appointments. Overall, nosocomial transmission accounted for 15% of closely genetically related (<2 SNPs) *C. difficile* ribotype 078 cases in this study, and equal proportions were attributable to farms and unknown transmission routes. In a study in Leeds, UK, which had comparable phylogenetic analysis, hospital ward-based epidemiologic linkage was reported as 11% for ribotype 078 cases versus 64% for ribotype 027 cases (*13*).

A EUCLID isolate from Ireland (2013) forms a genomic cluster with 1 CA-CDI isolate (2015) and 2 HA-CDI isolates (July 2015 and December 2015). These 4 isolates were from patients in 3 Dublin healthcare facilities and from 1 case of CA-CDI that had been collected within a 3-year period. This finding suggests shared exposure across the greater Dublin area, and that nosocomial transmission is not the dominant route of acquisition of *C. difficile* ribotype 078. This observation is consistent with the EUCLID study findings (*8*).

It is not clearly understood how persons who have CA-CDI acquired their infection because they do not have the risk factors for HA-CDI (*14*). Anderson et al. described proximity to livestock farms, agricultural industry, and nursing home facilities as risk factors for CA-CDI in North Carolina, USA, but they did not include analysis of *C. difficile* molecular data in their models (*15*). In contrast, Van Dorp et al. found no evidence of either localized point sources or livestock exposure as risk factors for *C. difficile* acquisition in the Netherlands (*16*). They included ribotype detail in their analysis, but found no evidence of geographic clustering of ribotype 078 CDI cases (*16*). This finding is consistent with that of Knetsch et al., who reported clonal isolates of farm and clinical origin without a geographic basis for those clusters (*10*).

Knetsch et al. identified another genomic cluster of *C. difficile* ribotype 078 isolates, which included an isolate of animal origin from Canada (2004) and 8 isolates of clinical origin from the United Kingdom (2008–2012) (*17*). We also identified a cluster of clinical and porcine 078 isolates from Ireland, where there was no known occupational exposure of the affected patients who lived in urban locations far from relevant pig farms. Knight et al. reported clonal ribotype 014 isolates from Australia that were considerable geographic distances from each other, which is suggestive of long-range transmission and major community reservoirs (*18*). They concluded that this transmission was unlikely to have been caused by direct contact between the humans and animals involved, and suggested that by-products, such as manure or compost, could enable indirect transmission from animals and humans (*18*). In a study from the United States, biosolid-based compost had the highest rate of *C. difficile* recovery that included ribotype 078 isolates (*19*), which was also the most common ribotype in an investigation of manure from Japan (*20*).

Findings based on ribotype analysis alone are insufficient for clear identification of transmission events pertaining to community reservoirs (*21*). Moradigaravand et al. identified ≈90% of their collection of clinical and wastewater isolates as clade 1 (231/256), and only 10 (3.9%) as clade 5/ribotype 078 (*22*). When their ribotype 078 isolates were compared with the same isolates from the Netherlands included in our analysis, they found divergence of ≈20 years between the isolates from the United Kingdom and the Netherlands. This finding suggests that water is not the primary reservoir or route for dissemination of *C. difficile* ribotype 078 isolates. It is still considered possible that dissemination of ribotype 078 isolates occurs by the food chain, the environment, or both (*23**,**24*). This view is supported by the presence and distribution of tetracycline-resistant determinants in *C. difficile* genomes, reflecting the antimicrobial drug selection pressure from tetracycline use in agriculture or veterinary practice, and thereby facilitating emergence and spread of ribotype 078 bacteria (*24*).

It is not completely understood how some livestock might have asymptomatic *C. difficile* colonization, whereas others show development of infection (*25*). The porcine isolates from Ireland in this analysis were from available samples processed at the CVRL. These isolates included samples from neonatal piglets that had typhlocolitis (*9*). We have identified genomic similarities among isolates causing human and veterinary infections. This finding augments the need for a One Health approach for *C. difficile* ribotype 078.

The strengths of this analysis include the large number of *C. difficile* ribotype 078 isolates included, from different sources including humans and animal species, and geographic origin. The limitations of this study include the lack of epidemiologic data available to the investigators for CA-CDI and the limited number of porcine strains from samples available at the CVRL. In conclusion, our analysis of *C. difficile* ribotypes 078 isolates from Ireland and 9 other countries in Europe showed close overlap between isolates from humans and pigs, including the occurrence of plausible transmission, either directly or by an unknown intermediate source.

Appendix Additional information on human and porcine transmission of *Clostridioides difficile* ribotype 078, Europe.
