# Supplementary material for: Human and Porcine Transmission of Clostridioides difficile Ribotype 078, Europe
Source: Emerg Infect Dis. 2021 Sep;27(9):2294–300. doi: 10.3201/eid2709.203468 (PMC8386809; doi:10.3201/eid2709.203468)
Supplement: Appendix — Additional information on human and porcine transmission of Clostridioides difficile ribotype 078, Europe. [file 20-3468-Techapp-s1.pdf]

Article DOI: https://doi.org/10.3201/eid2709.203468

Human and Porcine Transmission of *Clostridioides difficile* Ribotype 078, Europe

Appendix

Supplementary Methods

Mapping and Variant Calling

Reads were mapped by using Stampy version 1.0.23 (https://www.well.ox.ac.uk/research/research-groups/lunter-group/lunter-group/stampy) without Burrows-Wheeler Aligner premapping by using an expected substitution rate of 0.01. Samples were compared by using single-nucleotide polymorphisms (SNPs) identified with Samtools mpileup version 1.4.1 (www.htslib.org/doc/1.4.1/samtools.html) with the extended base-alignment quality flag. Python scripts with inputs from Samtools, Genome Analysis Toolkit version 3.7.0 (https://gatk.broadinstitute.org), Picard tools version 1.123 (https://broadinstitute.github.io/picard/), and vcftools version 0.1.9 (https://vcftools.github.io/index.html) were used to generate annotated variant call format files and for subsequent quality filtering. Filters included requiring an SNP quality score ≥25, a per base mapping score ≥30, a consensus ≥90% to support a SNP, and calls were required to be homozygous under a diploid model. Only SNPs supported by ≥5 reads, including 1 in each direction were accepted. SNPs were not called in repetitive regions of the genome identified by BLAST (https://blast.ncbi.nlm.nih.gov) to search for repeat regions >100 bp in length. Filtering rules were based on previous sequencing of technical replicates of bacterial genomes by using the same DNA pool (e.g., in Eyre et al. [*1*]), including visual inspection of alignments and chosen to keep the false-positive SNP rate to ≈1/100 Mb of genome sequenced. A containerized implementation of the pipeline used is available (https://github.com/oxfordmmm/CompassCompact).

Sequence Comparisons

Sequences in which <70% of the reference sequence was mapped were excluded from the analysis. To improve computational efficiency in identifying closely related sequences,

sequences within ≤500 SNPs of any other sequence were initially pooled into groups. For each group of sequences within ≤500 SNPs, initial maximum-likelihood phylogenetic trees were constructed by using PhyML version 3.0 (http://www.atgc-montpellier.fr), a generalized time-reversible substitution model, and the BEST tree topology search operation option. These trees were then adjusted to remove unrecombining regions by using ClonalFrameML version 1.25 (https://github.com/xavierdidelot/ClonalFrameML) and default parameters. Each recombination adjusted phylogenetic tree was used to determine the number of SNPs between all pairs of sequences (i.e., the patristic distance between them). An example implementation of this approach is available (https://github.com/davideyre/runListCompare).

Reference

1. Eyre DW, Cule ML, Wilson DJ, Griffiths D, Vaughan A, O'Connor L, et al. Diverse sources of *C. difficile* infection identified on whole-genome sequencing. N Engl J Med. 2013;369:1195–205. PubMed https://doi.org/10.1056/NEJMoa1216064
